# Navigating 3D electron microscopy maps with EM-SURFER

**DOI:** 10.1186/s12859-015-0580-6

**Published:** 2015-05-30

**Authors:** Juan Esquivel-Rodríguez, Yi Xiong, Xusi Han, Shuomeng Guang, Charles Christoffer, Daisuke Kihara

**Affiliations:** 1 Department of Computer Science, Purdue University, West Lafayette, IN 47907 USA; 2 Department of Biological Sciences, Purdue University, West Lafayette, IN 47907 USA; 3 Department of Mathematics, Purdue University, West Lafayette, IN 47907 USA

**Keywords:** Electron microscopy, Electron density maps, EM Data Bank, EMDB, 3D Zernike Descriptors, Proteins, Macromolecular structure, Low-resolution structure data, Database search

## Abstract

**Background:**

The Electron Microscopy DataBank (EMDB) is growing rapidly, accumulating biological structural data obtained mainly by electron microscopy and tomography, which are emerging techniques for determining large biomolecular complex and subcellular structures. Together with the Protein Data Bank (PDB), EMDB is becoming a fundamental resource of the tertiary structures of biological macromolecules. To take full advantage of this indispensable resource, the ability to search the database by structural similarity is essential. However, unlike high-resolution structures stored in PDB, methods for comparing low-resolution electron microscopy (EM) density maps in EMDB are not well established.

**Results:**

We developed a computational method for efficiently searching low-resolution EM maps. The method uses a compact fingerprint representation of EM maps based on the 3D Zernike descriptor, which is derived from a mathematical series expansion for EM maps that are considered as 3D functions. The method is implemented in a web server named EM-SURFER, which allows users to search against the entire EMDB in real-time. EM-SURFER compares the global shapes of EM maps. Examples of search results from different types of query structures are discussed.

**Conclusions:**

We developed EM-SURFER, which retrieves structurally relevant matches for query EM maps from EMDB within seconds. The unique capability of EM-SURFER to detect 3D shape similarity of low-resolution EM maps should prove invaluable in structural biology.

## Background

The three dimensional (3D) structure of proteins and other biomolecules provides the molecular basis for understanding mechanisms of biological functions, interactions, pathways, and serves as foundation for numerous areas in biotechnology. In addition to the exponential growth of solved 3D protein structures and complexes in the Protein Data Bank (PDB) [1,2], which are mostly determined by X-ray crystallography or NMR, low-resolution biomolecular structural data determined by cryo-electron microscopy (cryo-EM) and electron tomography are notably being rapidly accumulated in the Electron Microscopy Data Bank (EMDB, http://www.emdatabank.org/) [3]. Cryo-EM is an important technique in structural biology used to solve large protein complex and subcellular structures. Currently, EMDB holds over 2600 entries, and the number of entries is growing rapidly. The mean resolution of the EM maps is currently about 15 Å, but recent papers [4–6] report high-resolution structures at around 3.5 Å. There is no doubt that EMDB will become increasingly important not only in structural biology, but also in various areas including molecular biology and bioinformatics.

To take full advantage of these valuable resources of 3D biomolecular structures, it is necessary for one to be able to efficiently perform a structure-based search against the entire structure databases in real-time. Similarity search is the most essential operation that needs to be provided with a database. However, compared to biological sequence databases that are usually equipped with real-time database search methods, structure databases are behind with respect to efficient search methods, particularly for low-resolution structural data.

To this end, we have developed EM-SURFER for real-time searching of EM density maps from EMDB. Users can search for similar EM maps in EMDB in terms of the global shape and the volume of a query map. A query can be either chosen from existing EMDB entries or uploaded. Unlike atomic detailed structures stored in PDB, EM density maps are at low resolution and thus conventional structure comparison approaches cannot be directly applied.

A fast map comparison is achieved by using a mathematical representation of 3D shapes named 3D Zernike Descriptor (3DZD) [7]. 3DZD is a vector derived from a series expansion of a 3D function, which describes an EM map in a compact and rotation-invariant fashion. 3DZD has been successfully applied to represent various biomolecular structure analyses [8], including protein 3D shape comparison [9], protein docking [10–12], ligand binding site comparison [13,14], and fast ligand database search [15].

In EM-SURFER, each search is performed on-the-fly and only takes a few seconds. The database of EM maps is automatically synchronized with EMDB weekly. In what follows, we first describe how 3D EM maps are represented in EM-SURFER, and then explain input data and output search results with examples.

## Implementation

The main operation performed by EM-SURFER involves comparing two EM maps using an efficient structure representation with 3DZD. The descriptor is derived from a mathematical series expansion of a 3D function based on the 3D Zernike moments. 3DZD was originally derived by Canterakis [7] and later applied to 3D object retrieval [16]. A 3DZD can be viewed as a fingerprint that consists of a vector of real numbers, where each number is a coefficient of the series expansion. Comparisons between these fingerprints form the basis of the rapid search performed by our server. The similarity between 3DZD vectors is quantified by their Euclidean distance.

EM density maps for EM-SURFER are obtained from EMDB [3], the primary repository of electron microscopy data, and updated on a weekly basis. For each EM map, 3DZD vectors are computed. It was shown in previous studies [17,18] that 3DZD can properly represent EM maps. An EM map is a 3D grid where an electron density value is assigned at each grid point. Using the author-recommended density contour level provided in EMDB, grid points with an electron density that is equal or larger than the author-recommended density are marked with 1 and 0 otherwise. The value-mapped 3D grid was considered as a 3D function, *f(x)*. This *f(x)* is expanded into a series in terms of the Zernike-Canterakis basis defined as follows:

$$ {\varOmega}_{nl}^m=\frac{3}{4\pi }{\displaystyle {\int}_{\left|\mathbf{x}\right|\le 1}f\left(\mathbf{x}\right){\overline{Z}}_{nl}^m\left(\mathbf{x}\right)d\mathbf{x}} $$

where

$$ {Z}_{nl}^m\left(r,\vartheta, \phi \right)={R}_{nl}{Y}_l^m\left(\vartheta, \phi \right) $$

The ranges of parameters *l* and *m* are defined by the order *n*: − *l* < *m* < *l*, 0 ≤ *l* ≤ *n*, and *n-l* even. We used order n = 20, which corresponds to 121 invariants. ${Y}_l^m\left(\vartheta, \phi \right)$ are the spherical harmonics and *R*_*nl*_(*r*) are the radial functions constructed in a way that ${Z}_{nl}^m\left(r,\vartheta, \phi \right)$ can becalculated as norms of vectors Ω^m^_nl_. The norm gives rotational invariance to the descriptor:

$$ {F}_{nl}=\sqrt{{\displaystyle \sum_{m=-l}^{m=l}{\left({\varOmega}_{nl}^m\right)}^2}} $$

A similar rotation-invariant 3D shape descriptor can be constructed by using only spherical harmonics ${Y}_l^m\left(\vartheta, \phi \right)$. Particularly, in the spherical harmonics descriptor (SHD), a 3D object is segmented by a set of concentric spheres, for each of which a rotation-invariant descriptor using spherical harmonics is constructed and concatenated to incorporate distance information from the object center [19–21]. 3DZD is mathematically superior to SHD because SHD computes rotation invariant descriptor for each concentric sphere separately, and thus the shells can be rotated independently by random angles without changing the resulting descriptors. Also, in 3DZD, the orthonormality of the Zernike-Canterakis basis results in less information redundancy. In contrast, in SHD, descriptors coming from adjacent shells are highly correlated, making them redundant to some extent. That usually makes the size (the length of the descriptor) of SHD larger than 3DZD. Moreover, 3DZD was shown to perform better than SHD in shape-based object retrieval [16] and protein global surface shape comparison [22]. For more discussion about 3DZD and spherical harmonics, refer to a review paper [23].

The distance between two 3DZDs is quantified as the Euclidean distance between the vectors. Comparisons between fingerprints form the basis of the rapid search performed by our server. A more detailed derivation of 3DZD as well as the mathematical foundation can be found in previous publications [7,16,24].

**Figure 1:**
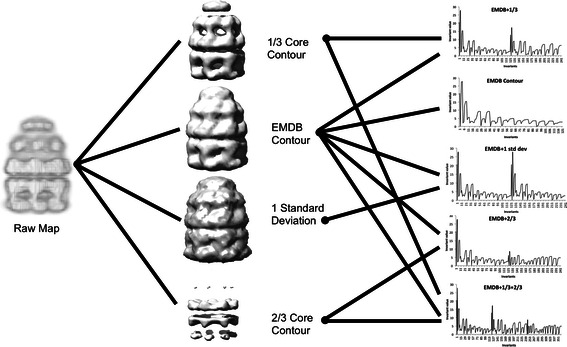
3DZD computation pipeline. Every map in EMDB yields several 3D Zernike descriptor fingerprints. The raw map is used to generate four voxelizations: one from the author-recommended density value, one at one standard deviation, which is lower than the author-recommended contour level, and two additional thresholds that reveal core features. Each surface is represented by 121 descriptors, which are concatenated to generate various fingerprints.

Besides the author-recommended density level, a voxelization at one standard deviation of electron density, and two additional voxelizations at higher density levels, 1/3 and 2/3 of the highest density, were computed (Figure 1). The purpose of the additional map descriptions with one lower and two higher densities is to capture shapes at different contour levels of the molecules. Each contour level yields its own vector of 121 3DZD invariants. In total, five EM map descriptors were prepared: the 3DZD for 1) the author-recommended density level, descriptors that concatenate the 3DZD of 2) the author-recommended density level and another 3DZD computed at one standard deviation, 3) 1/3 maximum density, or 4) 2/3 maximum density, and 5) a descriptor that concatenates the author-recommended and 1/3 and 2/3 density level 3DZDs. The second to the fourth descriptors have 242 invariants and the last one has 363 invariants. The 3DZDs were pre-computed for each EMDB entry. They will be computed on-the-fly for a query if users upload their own EM map.

PDBj (Protein Databank Japan, http://pdbj.org/) provides a list of structurally similar maps for each EM map entry in their EM Navigator. Similar maps are identified by vector quantization and the similarity of all EM maps are visualized in a two dimensional map (named the Omokage map) computed by multidimensional scaling. Although details of the implementation of the method are not provided at the EM Navigator website (http://pdbj.org/emnavi/emnavi_doc.php?doc=omokage), differences between EM-SURFER and EM Navigator include the following: Unlike in the Omokage map, which seems to be pre-computed, similarity search for a query is performed on-the-fly in EM-SURFER. Thus, a search can be performed also for a map that is uploaded by a user.

The validity of applying 3DZD for EM map database search was shown in previous studies [17,18]. These two studies demonstrated database searches for simulated and actual EM maps, which achieved high accuracy by describing EM maps with 3DZD.

## Results and discussion

**Figure 2:**
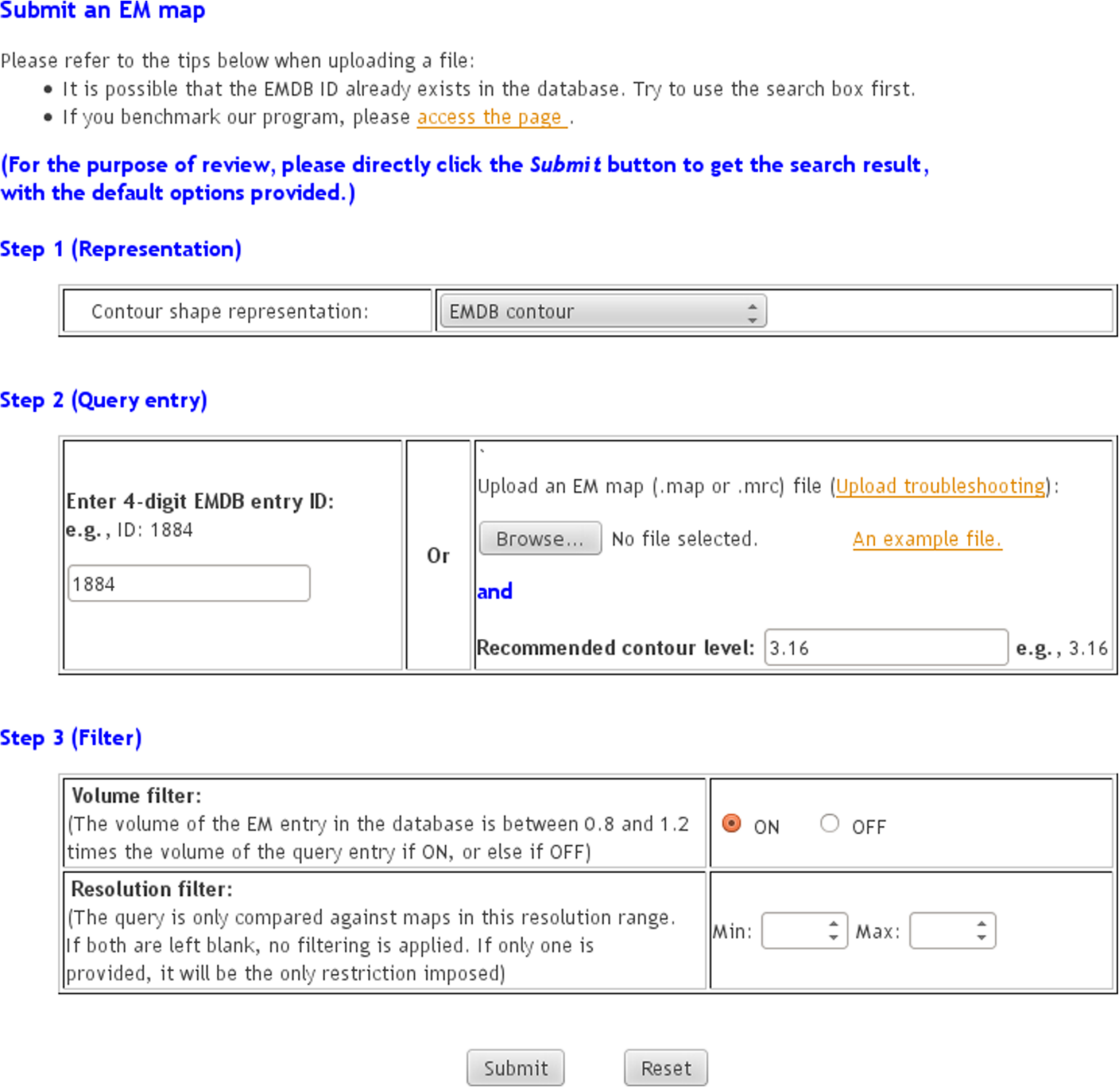
EM-SURFER query submit page. There are three steps for query submission. See text for details.

The main result generated by EM-SURFER is a list of EM maps, with queries submitted through the Search page (Figure 2). To submit a query entry, users should go through the following four steps. In Step 1, the contour shape representation should be specified. The default is set to the author-recommended contour level. In Step 2, users need choose the EMDB entry ID or upload an EM map file. To find an ID from a protein name or other information, use the EMDB text search page at http://www.ebi.ac.uk/pdbe/emdb/searchForm.html. In Step 3, a volume filter is provided, which is enabled by default. When this filter is on, a search only retrieves EM maps that have a volume similar to the query (the ratio between the query and each retrieved map should be between 0.8 to 1.2). Finally, a resolution filter allows users to restrict the maps returned for the query to be in the specified resolution range.

**Figure 3:**
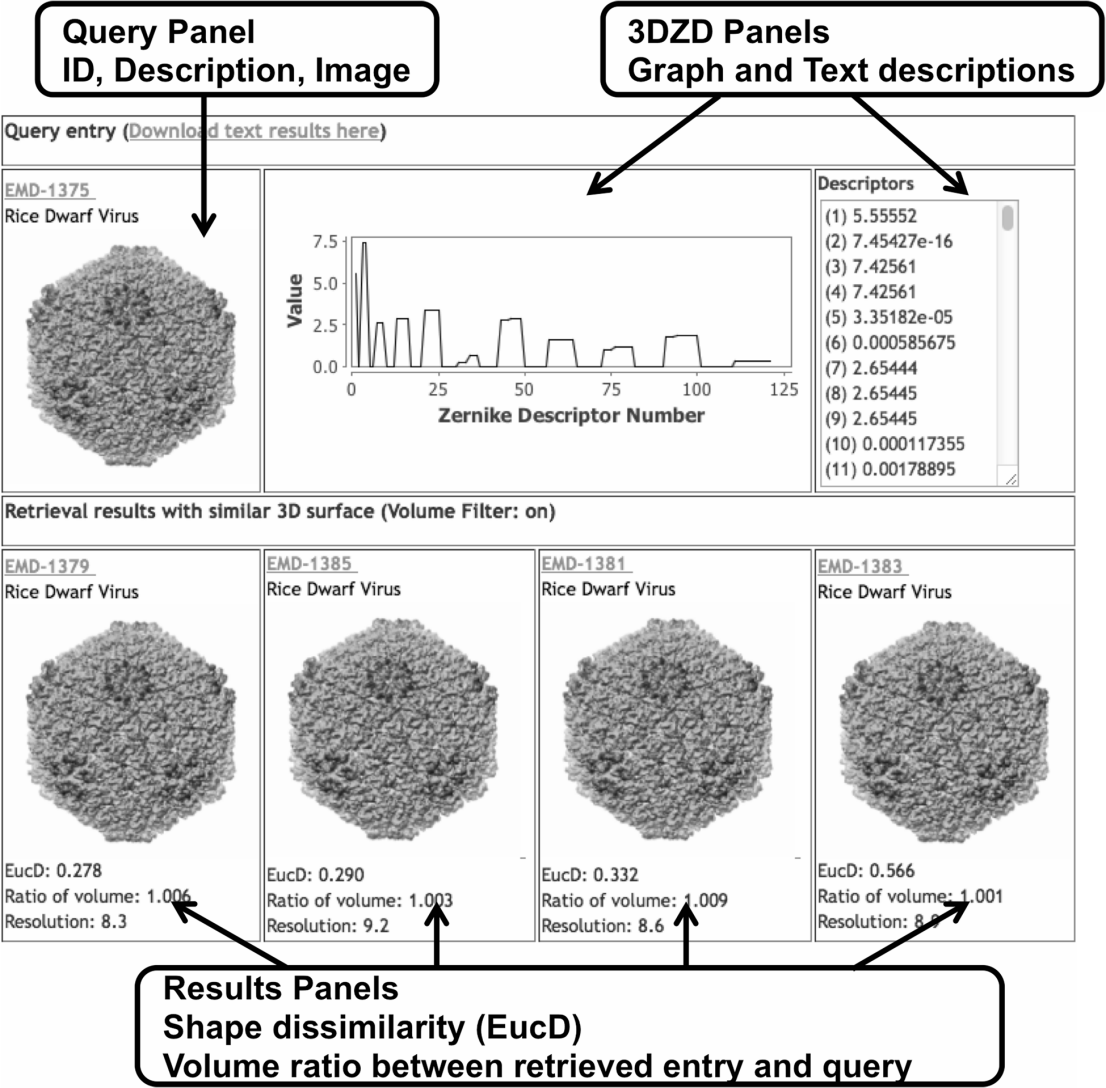
EM-SURFER results sections. EMD-1375 was used as a query to explain the different sections returned by EM-SURFER. On the top-left section, an image of the original query is shown (or the filename if it was a user-uploaded query). Beside this image, a graphical and text depiction of the 3D Zernike descriptors that describe the map are shown. Finally, for each search hit it shows the EMDB code with a short description, an image, and the detailed values for Euclidean distances, volume ratio and resolution.

The results page displays the top 20 entries in the database that have the most similar global shape to the query EM map. Figure 3 shows the four most similar EM maps for EMD-1375 as query. In the top panel, it shows the query entry ID and its molecule name, a figure of the query (which is provided by EMDB), as well as the 3DZD that characterizes the query entry in text and graphic forms. The query entry ID is a unique 4-digit accession number used in EMDB. Also in the top panel, the user is given a link to a text file for a list of the most similar maps. In the bottom graphic panel, a list of retrieved entries for the query is shown. They are ranked by the distance of their 3DZDs to that of the query entry (quantified by Euclidean distance, EucD, i.e. the square root of the sum of the squares of the differences between corresponding values). The smaller the EucD is, the more similar the shapes of the two EM maps are. Empirically, entries with a Euclidean distance of less than 8.0 are biologically related. For each retrieved entry, it also shows the ratio of the volume of the retrieved entry to the query, which is defined as the volume of the retrieved entry divided by that of the query, as well as the resolution of the map. Clicking on the image of a retrieved entry will trigger a new search using the clicked entry as a query.

**Figure 4:**
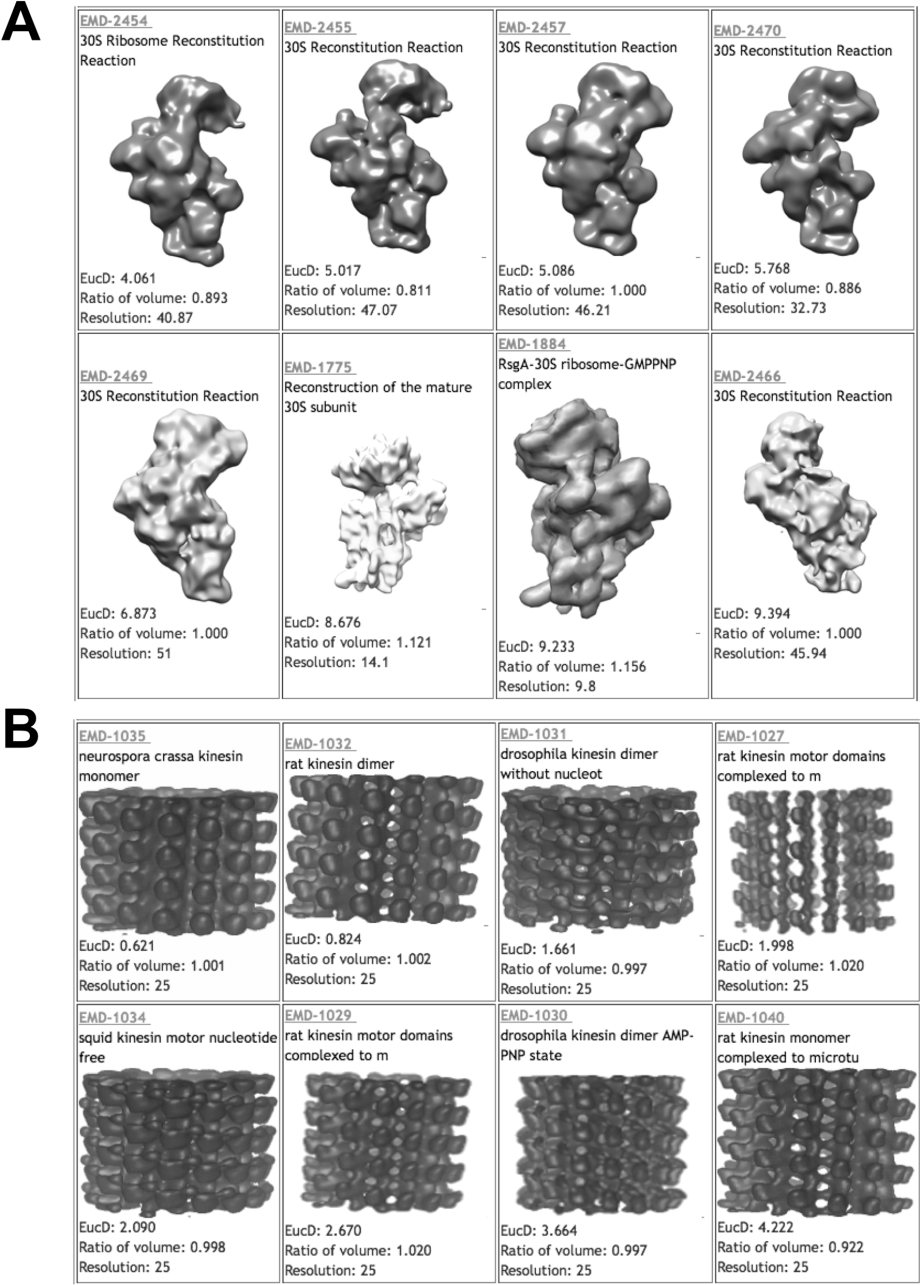
Example search results in EM-SURFER. Two examples of search results by EM-SURFER. Query maps used were **A**, 30S ribosome subunit assembly (EMD-2456) and **B**, tubulin with bound dimeric kinesins (EMD-1033). The top 8 hits are shown.

We show three examples of search results by EM-SURFER. For these searches, the author-recommended density level was used. Only structures with a resolution provided in their meta-data are retrieved in these examples. The volume filter was on. In Figure 4 and Table 1, detailed information of the top eight most similar EM maps for the first two queries are shown. The first example is a search from a 30S ribosomal complex structure (EMD-2456). Among the top 10 most similar maps retrieved from the database, all of them are 30S ribosomal subunit structures. The second example (Figure 3B) shows search results of tubulin that have cylindrical-shape (EMD-1033). The top thirteen retrieved EM maps are all from tubulins. Similar to the first example, entries retrieved with a Euclidean distance of 6.5 or less are all tubulins. The second example demonstrates that EM-SURFER can retrieve similar EM maps not only for globular-shape EM maps but also for cylindrical complexes.

**Table 1:** **Search results of the two queries by EM-SURFER**

**Rank**	**EMDB ID**	**Volume Ratio**	**Distance**	**Molecules**
**EMD-2456 30S ribosomal complex**				
1	2454	0.893	4.061	30S ribosome
2	2455	0.811	5.017	30S ribosome
3	2457	1.000	5.086	30S ribosome
4	2470	0.866	5.768	30S ribosome
5	2469	1.000	6.873	30S ribosome
6	1775	1.121	8.676	30S subunit
7	1884	1.156	9.233	30S ribosome- GMPPNP
8	2466	1.000	9.394	30S ribosome
**EMD-1033 tubulin with bound dimeric kinesins**				
1	1035	1.001	0.621	Tubulin
2	1032	1.002	0.824	Tubulin
3	1031	0.997	1.661	Tubulin
4	1027	1.020	1.998	Tubulin
5	1034	0.998	2.090	Tubulin
6	1029	1.020	2.670	Tubulin
7	1030	0.997	3.664	Tubulin
8	1040	0.922	4.222	Tubulin

**Figure 5:**
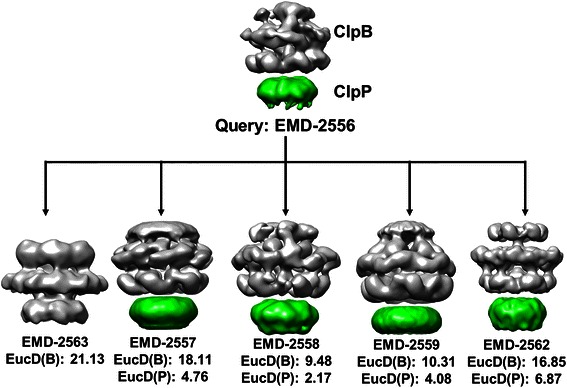
Similarity of EM maps of ClpB. ClpB (gray) and bound ClpP (green) in the query, EMD-2566, was separately compared against the corresponding part of four related entries, EMD-2563, 2557, 2558, 2559, and 2562. The numbers are the Euclidean distances of 3DZDs between them. EMD-2563 contains only ClpB. The Euclidean distance shown for EMD-2563 (21.13) is computed for the complete maps of EMD-2556 and EMD-2563.

The examples shown above demonstrate that EM-SURFER successfully retrieves related entries of the same molecules. However, since EM-SURFER performs global shape and volume comparison between EM maps, entries of the same molecule but in different conditions that lead to overall different shape would not be retrieved at a high rank, even if they would be easily retrieved by the text search, which is currently available at EMDB. Table 2 and Figure 5 provide results that exemplify this type of situation. Nine EMDB entries, EMD-2055 to 2563, are maps under different conditions and mutants of hexameric AAA+ chaperone ClpB (gray region in Figure 5) bound (or not bound) to protease ClpP (green). These entries were reported in the same paper [25]. Six copies of ClpB assemble into a ring-shape complex (gray region) and work as chaperone, where a misfolded protein will go through the pore at the center of the hexamer ring and be unfolded. In a study by Carroni et al., mutants of ClpB were constructed that lock the complex in active or repressed states, which yielded the nine EM structures [25].

**Table 2:** **Distance between entries of ClpB in various conditions**

**EMDB ID**	**Description** ^**a)**^	**Distance from 2556**
2556	ClpB E432A ATPγS. BAP variant bound to ClpP	0.00
2555	ClpB E432A ATPγS. BAP variant bound to ClpP	7.22
2557	ClpB ATPγS. BAP variant bound to ClpP	22.89
2558	ClpB ATPγS. BAP variant bound to ClpP	7.11
2559	ClpB Y503D mutant with ATPγS. BAP variant bound to ClpP	12.32
2560	ClpB Y503D mutant with ATPγS. BAP variant bound to ClpP	7.58
2561	Hsp104 ATPγS. HAP variant bound to ClpP	17.81
2562	ClpB DWB trap mutant with ATPγS. BAP variant bound to ClpP	21.70
2563	ClpB with ATPγS	22.75

a) Description was taken from the sample record of the entries in EMDB.

As shown in Table 2, when a search was performed from query EMD-2556, not all the other eight entries were close: Three entries, EMD-2555, 2558, and 2560, were retrieved within a distance of 8.0, but the remaining five entries (EMD-2557, 2559, 2561, 2562, and 2563) were more distant than 10.0 (12.0 to 23.0). To understand why the five entries have a large distance, we computed the similarity of ClpB (gray) and ClpP (green) regions separately (Figure 5). Interestingly, it turned out that actually those entries that have a large Euclidean distance have ClpB in different shapes reflecting their different functional states. The ClpP region is similar in all the entries (the distance ranges from 4.08 to 6.87). In the case of EMD-2563, it does not even have bound ClpP in the map, which makes the overall shape of the map completely different from the shape of the query. Thus, in this example, EM-SURFER detected different states of the same complexes, which would be very useful for analyzing sub-states of the same macromolecules.

The current EM-SURFER identifies entries with globally similar shape to the query EM map, but does not detect local shape similarity between maps. Local map similarity search is left as future work.

## Conclusions

We reported a web application named EM-SURFER for real-time biomolecular structure search based on electron microscopy density maps. EM density maps are updated weekly from EMDB. The unique feature of EM-SURFER, the ability of searching EM maps by shape similarity in a matter of seconds, should prove invaluable in structural biology. A similar strategy will be also valuable for other types of low-resolution biological structure data.

## Availability and requirements

**Project name:** EM-SURFER

**Project home page:** http://kiharalab.org/em-surfer

**Operating system(s):** Web application, platform independent
